# PET/CT with Fibroblast Activation Protein Inhibitors in Breast Cancer: Diagnostic and Theranostic Application—A Literature Review

**DOI:** 10.3390/cancers15030908

**Published:** 2023-01-31

**Authors:** Silvia Taralli, Margherita Lorusso, Elisabetta Perrone, Germano Perotti, Luca Zagaria, Maria Lucia Calcagni

**Affiliations:** 1 Nuclear Medicine Unit, Dipartimento di Diagnostica per Immagini, Radioterapia Oncologica ed Ematologia, Fondazione Policlinico Universitario A. Gemelli IRCCS, 00168 Rome, Italy; 2 Dipartimento Universitario di Scienze Radiologiche ed Ematologiche, Università Cattolica del Sacro Cuore, 00168 Rome, Italy

**Keywords:** PET/CT, 68Ga-FAPI, FAPI, breast cancer, theranostics

## Abstract

**Simple Summary:**

The fibroblast activation protein (FAP) is highly expressed on cancer-associated fibroblasts in many tumors. Radiolabeled FAP inhibitors (FAPIs) have been recently developed as new promising radiotracers in an oncological setting, both as diagnostic imaging by Positron Emission Tomography/Computed Tomography (PET/CT) and as new anti-cancer treatment, in a theranostic perspective. This narrative review aimed to summarize the current evidence on the role of FAPI radiotracers in the diagnostic and therapeutic management of patients with breast cancer (BC). Despite some clinical and methodological heterogeneity among the evaluated studies, ^68^Ga-FAPI PET/CT emerges as a valuable diagnostic tool in BC patients, demonstrating a general high tumor uptake across all histological and molecular BC subtypes, along with technical advantages and an overall better performance than the standard-of-care ^18^F-FDG. Moreover, although with still limited clinical evidence, treatment with radiolabeled FAPIs seems a promising approach in BC patients, increasing the possibility of more personalized treatments.

**Abstract:**

Growing studies have recently reported on the promising application of radiolabeled-fibroblast activation protein inhibitors (FAPIs) as diagnostic and therapeutic agents in various oncological populations. To exclusively evaluate the current evidence on the diagnostic and therapeutic role of FAPI radiotracers in patients with breast cancer (BC), a narrative review of the available literature was performed. A search algorithm from PubMed/MEDLINE, based on the combination of “PET” OR “positron emission tomography” and “FAPI” and ”cancer”, with a last update in February 2022, was applied. From 233 identified articles, 33 studies conducted in BC patients and with available data on PET imaging or radiolabeled-FAPI therapy were finally considered, for a total of 191 patients. Despite some clinical and methodological heterogeneity among the reviewed articles, ^68^Ga-FAPI PET/CT emerges as a valuable diagnostic tool in BC patients both at staging and restaging, also demonstrating several technical advantages and an overall better performance than ^18^F-FDG, especially in histotypes with well-known low ^18^F-FDG avidity. Moreover, although with still limited clinical evidence in BC, radiolabeled FAPIs emerge as promising therapeutic agents in a theranostic perspective, increasing the possibility of more personalized treatments. From these results, future research directions on FAPI radiotracers application in BC patients are suggested.

## 1. Introduction

Tumor microenvironment (TME), in addition to malignant cells, represents the main component of solid neoplasms. TME is composed by extracellular matrix (ECM) and various non-malignant cells such as fibroblasts and immune, precursor, mesenchymal stromal and endothelial cells that interact with tumor cells through signaling molecules, in a dynamic and complex balance between pro-tumoral and tumor-suppressive functions [1]. Among TME cells, cancer-associated fibroblasts (CAFs) are important constituents of the tumor stroma [2,3], being able to contribute to tumorigenesis by production of many growth factors and pro-inflammatory cytokines and chemokines [1]. In particular, through immunosuppressive activity and mediators’ productions, CAFs support tumor development and progression by promoting tumor cells proliferation, migration, invasion, angiogenesis and resistance to therapies [1,4,5]. Fibroblast activation protein (FAP), a type II transmembrane serine protease belonging to the dipeptidyl peptidase-4 family [6], is highly over-expressed on CAFs membrane in about 90% of epithelial-derived tumors (such as breast, stomach and pancreatic carcinomas), so representing a marker of CAFs activation. Due to their close association with tumor progression, metastatic spread and treatment resistance, CAFs and, in turn FAP, are recently emerging as promising targets for cancer diagnosis and treatment [4,7]. In this context, radiopharmaceuticals based on fibroblast activation protein (FAP)-specific inhibitors (FAPIs) have been recently developed, so emerging as new promising tracers for Positron Emission Tomography/Computed Tomography (PET/CT) imaging [8,9,10]. In particular, since 2018, researchers from the University of Heidelberg showed that DOTA-containing FAP inhibitors (FAPI) can be coupled with Gallium-68 and used for diagnostic PET imaging of multiple tumor entities, such as breast, colon, lung and pancreatic cancer [10,11]. Beside the use of radiolabeled-FAPI tracers for a diagnostic purpose, FAP-targeting ligands labeled with alpha- or beta-emitting radioisotopes (e.g., Lutetium-177, Yttrium-90, Actinium-225) have been also introduced, exploiting their potential role as a new anti-cancer radioligand therapy (RLT), in a theranostic perspective [10,12]. Up to date, most clinical oncologic studies on the diagnostic and/or theranostic application of FAPI radiotracers include patients with mixed tumors and heterogeneous clinical settings. In patients with breast cancer (BC), some promising results on the value of FAPI ligands for both diagnostic and therapeutic purposes have been reported. In particular, FAPI tracers seem to play a role in the diagnostic evaluation of primary BC and metastatic lesions, with some evidence on better diagnostic performance when compared to ^18^F-Fluorodeoxyglucose (^18^F-FDG) [13,14]. As rationale for the use of radiolabeled FAP-ligands in BC, it has been demonstrated that TME has a role in BC development, with the disease evolution depending not only on the intrinsic behavior of cancer cells (according to differences in gene expression patterns, hormonal receptor status, human epidermal growth factor receptor 2–HER2–expression, etc.), but also on TME composition and on the interactions between cancer cells and TME itself [15]. In this scenario, CAFs are highly represented in the BC microenvironment, so making FAP an excellent candidate as an indirect tumor cell target for both PET diagnostic imaging and RLT, as supported by a recent study that found a strong correlation between ^68^Ga-FAPI uptake and FAP tissue expression in several different cancer types, including BC [2]. Radiolabeled FAPIs have been suggested as a therapeutic target in recent years [8,12], using various radiopharmaceuticals for treatment, such as ^177^Lu-DOTAGA.(SA.FAPI), ^177^Lu-DOTA.SA.FAPi, ^225^Ac-FAPI-04, ^153^Sm-FAPI-46 and ^90^Y-FAPI-04 [9,16,17,18]. The alpha/beta labelled FAPIs can deliver a high radiation dose to destroy the tumor cells mainly by bystander effects [19]. For RLT application, the main criticism is the rapid wash-out of the radiotracer, which is completely eliminated by 48 h from injection. Recently, ^177^Lu-DOTAGA.(SA.FAPI)2 was developed and demonstrated a high tumor uptake and prolonged effective half-life, implying a higher adsorbed dose to the whole body (mainly to gallbladder, pancreas, kidneys and liver).

To the best of our knowledge, a comprehensive review exclusively focused on the application of radiolabeled-FAPI tracers in BC patients is still lacking. So, the purpose of this narrative review is to summarize the available evidence regarding the role of FAPI radiotracers in the management of BC patients.

## 2. Materials and Methods

### 2.1. Literature Search

A comprehensive computer literature search of the PubMed/MEDLINE database was conducted in order to find relevant published articles on the use of radiolabeled FAPI in patients with BC, including both their application as PET diagnostic imaging and as radioligand therapy. A search algorithm based on the combination of the terms (“PET” OR “positron emission tomography”) AND (“FAPI”) AND (“cancer”) was used. The last update of the literature search was 15 February 2022. Titles and abstracts of the retrieved studies were screened independently by two researchers (ST and ML).

### 2.2. Inclusion and Exclusion Criteria and Data Analysis

Only articles written in the English language and studies conducted on human subjects, that included patients affected by BC, and with available data on PET imaging or radionuclide therapy with radiolabeled-FAPI (regardless of disease stage and clinical indication for diagnostic or therapeutic procedures) were considered. After screening titles and abstracts, an initial selection was performed excluding: (a) articles not within the field of interest of this review (e.g., articles not evaluating patients with BC; focused on FAPI synthesis and biodistribution; with no data on imaging or therapy); (b) pre-clinical studies; (c) review articles and meta-analyses, editorials, letters, commentaries and conference proceedings. Then, two researchers (ST and ML) independently reviewed the full-text version of the selected articles for final eligibility. Disagreements were resolved by consensus. For each included article, information was collected about basic clinical characteristics (study design, number of BC patients, clinical indication, etc.), and methodological aspects (FAPI tracer, injected tracer activity, scan delay defined as the time interval between tracer injection and PET image acquisition, comparison with ^18^F-FDG PET imaging (if available), etc.).

## 3. Results

### 3.1. Literature Research

From the initial literature research, 233 articles were retrieved. Applying exclusion and inclusion criteria, 33 articles reporting on radiolabeled FAPI application in at least one patient with primary BC were identified, with data on BC mostly extracted from a mixed population of patients with various cancers. In details: 27/33 articles exclusively evaluated FAPI PET diagnostic imaging [2,6,8,10,11,12,20,21,22,23,24,25,26,27,28,29,30,31,32,33,34,35,36,37,38,39,40]; 6/33 reported theranostic FAPI application in a total of 16 metastatic BC patients [9,25,41,42,43,44]. These 33 articles have been fully read and analyzed and their main results are herein summarized.

### 3.2. Diagnostic PET Imaging

#### 3.2.1. Clinical and Methodological Studies’ Characteristics

Table 1 reports the main clinical and methodological characteristics of the 27 studies evaluating FAPI PET in BC patients. In detail: 20/27 were case reports or included data derived from no more than four patients; in 4/27, the cohort’ size ranged from 12 to 20 BC patients, with data extracted from a mixed population of various cancers [6,11,25,31]; only 3/27 studies evaluated a larger and exclusively BC population, ranging from 19 to 48 patients [29,32,39]. Regarding study design, most of the studies were prospective or presented a retrospective analysis in patients already enrolled in a prospective trial. PET with FAPI tracers was performed for two main clinical indications: staging in newly diagnosed BC patients and restaging for clinic-radiological suspicious of recurrent/metastatic disease in pre-treated patients. In all studies, the imaging modality was PET/CT, except for the study by Backhaus et al. [39] that performed an additional breast Positron Emission Tomography/Magnetic Resonance (PET/MR) in 18/19 patients and a whole-body PET/MR in half of patients. Among several developed FAPI radiotracers, all studies used ^68^Gallium-labeled FAPI, ^68^Ga-FAPI-04 being the most frequently applied (>60%), followed by ^68^Ga-FAPI-02 and ^68^Ga-FAPI-46. The PET/CT scan was generally acquired 60 min after administration of a mean tracer activity of 1.8–2.2 MBq/kg, with a field of view extended from vertex to upper/mid-thighs. Regarding PET images analysis, the most commonly used semiquantitative parameters were lesions’ maximum and mean Standardized Uptake Values (SUVmax and SUVmean); moreover, tumor-to-background ratio (TBR) was also measured, with different tissues selected as background reference (liver, lung, bone, blood pool, muscle, etc.).

#### 3.2.2. ^68^Ga-FAPI PET Diagnostic Performance: Primary BC Lesion and Comparison with ^18^F-FDG

Five studies specifically evaluated and reported the primary breast lesion activity, using ^68^Ga-FAPI-04, ^68^Ga-FAPI-46 or ^68^Ga-DOTA.SA.FAPI. At a mean of 30 to 60 min after tracer injection, a variable but generally high ^68^Ga-FAPI uptake was observed, resulting in a clear primary tumor lesions’ delineation, with mean and median SUVmax values ranging from 10.0 to 16.4 [29,32,39], and from 8.4 to 17.4, respectively [29,31], and with an average SULpeak (i.e., Standardized Uptake Value corrected for lean body mass) of 6.5 ± 3.3 using ^68^Ga-DOTA.SA.FAPi [25]. Although some differences in measured BC activity were also observed according to different tumor histotypes, hormonal receptors expression (estrogen receptor (ER) and progesterone receptor (PR)) and grading, the tumor histological characteristics seem not or only partially accountable for the variations in primary BC activity. In detail, in a population of 19 BC patients, a higher but not statistically significant ^68^Ga-FAPI uptake was described by Backhaus et al. in ductal cancers (*n* = 16) compared to lobular cancers (*n* = 3), with a median SUVmax of 14.5 and 10.6, respectively (*p* = 0.20). The same authors did not find statistically significant differences in ^68^Ga-FAPI activity among the three main BC molecular subtypes (ER-positive HER2-negative, HER2-positive, triple-negative), observing similar values of mean SUVmax [39]. Conversely, when further stratifying BC molecular subtypes in 5 classes (Luminal A, Luminal B HER2-negative, Luminal B HER2-positive, HER2-enriched and triple-negative), Elboga et al. showed a trend for increasing SUVmax values from Luminal A (lower values) to Luminal B HER2-positive type and HER2-enriched tumors (higher values); moreover, HER2 expression seems to confer the highest ^68^Ga-FAPI activity among the Luminal group, with significantly higher uptake in Luminal B HER2-positive vs. Luminal A or Luminal B HER2-negative groups [32]. With respect to tumor grading, Dendl et al. observed a stronger ^68^Ga-FAPI uptake in high-grade tumors compared to low-grade ones, although in a mixed population of gynecological malignancies (BC = 14) and with no statistical relevance [6]. Besides, neither did Backhaus et al. find any significant difference in ^68^Ga-FAPI uptake when considering tumor grading in 19 patients with BC [39]. With respect to the possible influence of BRCA 1/BRCA 2 genes pathogenic mutations over ^68^Ga-FAPI, very limited data are available: Dendl et al., in their mixed population of BC and other gynecological malignancies, observed a slightly higher uptake in BRCA 1/2 positive patients (*n* = 6) than in patients without mutations (*n* = 6), although this difference was not statistically significant [6]. When comparing the diagnostic performance of ^68^Ga-FAPI and ^18^F-FDG in detecting primary breast lesions (Table 2), some discrepant and variable findings were reported in the literature. Indeed, cases of BC with absent ^18^F-FDG activity and intense ^68^Ga-FAPI uptake can be found [34], but also studies reporting comparable BC activity between the two radiotracers (SULpeak range: 3.3–12.5 for ^68^Ga-FAPI, and 1.2–16.9 for ^18^F-FDG), so concluding that the diagnostic accuracy of the newly introduced ^68^Ga-FAPI seems similar to the standard-of-care ^18^F-FDG [25]. However, most of the literature evidence reported a significantly higher tracer uptake and higher TBR of primary BC with ^68^Ga-FAPI radiotracers, in comparison to ^18^F-FDG, so agreeing that BC is one of the tumor entities with a better detection rate at ^68^Ga-FAPI PET/CT [45]. In particular, such results were demonstrated by Elboga et al., who observed higher values of ^68^Ga-FAPI uptake, regardless of BC histotype (ductal or lobular carcinoma) [32]. Interestingly, the authors found higher SUVmax values with ^68^Ga-FAPI than ^18^F-FDG for invasive lobular cancer (ILC), which usually shows low glycolytic metabolism, although without achieving statistical significance due to the low size of the ILC-group. Moreover, higher SUVmax and TBR values with ^68^Ga-FAPI-04 over ^18^F-FDG were confirmed by Komek et al., who also reported a higher ^68^Ga-FAPI sensitivity for detecting the primary tumor, with similar specificity: sensitivity and specificity of 100% and 95.6% for ^68^Ga-FAPI, and of 78.2% and 100% for ^18^F-FDG, respectively [29]. Finally, a significantly higher uptake for ^68^Ga-FAPI over ^18^F-FDG was recently reported also in a case of inflammatory BC (median SUVmax of 28.9 vs. 10.1, respectively) [35].

#### 3.2.3. Physiological ^68^Ga-FAPI Breast Uptake

When considering physiological breast tissue activity, a variable but generally low ^68^Ga-FAPI uptake is observed in healthy mammary parenchyma, with a mean SUVmax of 1.1 (±0.5) in the normal breast in the largest cohort of 147 female patients evaluated by Dendl et al. [2,6,39]. This finding is consistent with the little or no FAP expression assessed by immunohistochemistry in normal breast tissue [32]. Only in a few cases has a relatively increased radiolabeled-FAPI uptake been observed in the normal breast, with a maximum SUVmax of 6.9 and a mean SUVmax of 2.5 in a study population of 19 BC patients [39], and a mean SUVmax of 4.5 ± 1.5 in 7.7% of a miscellaneous oncologic population [4]. Nevertheless, this functional occurrence does not seem to affect PET performance for detecting BC lesions, since a still favorable tumor-to-background ratio was observed, so providing a sufficient tumor delineation [39].

#### 3.2.4. Effect of Hormonal Status on ^68^Ga-FAPI Breast Uptake

It should be considered that FAP expression in hormone-sensitive organs, such as endometrium and breast, seems to be modulated by endogenous and exogenous hormonal stimulation, translating into a variable degree of ^68^Ga-FAPI uptake. In particular, according to literature evidence, increased FAPI tracer uptake in the endometrium and breast is generally observed under hormonal stimulation, likely due to up-regulation of tissue FAP expression induced by higher estrogen levels [46]. In this regard, in a large cohort of 167 female patients with various malignancies, Dendl et al. specifically analyzed ^68^Ga-FAPI uptake in normal hormone-responsive organs: breast (*n* = 147 pts), endometrium (*n* = 128 pts) and ovary (*n* = 64 pts), classifying the patients by age in: pre-menopausal (<35 years), post-menopausal (>65 years) and with unknown menstrual status (35–65 years), respectively [6]. They found significant differences in breast and endometrium ^68^Ga-FAPI uptake between the pre- and post-menopausal status, with no differences in ovarian activity. In particular, a significantly higher tracer uptake in breast (mean SUVmax 1.8 vs. 1.0; *p* = 0.004) and endometrium (mean SUVmax 11.7 vs. 3.0; *p* < 0.001) was found in pre-menopausal patients, compared to post-menopausal ones. The authors argued that higher endometrial uptake in pre-menopausal women might be due to cyclic endometrial regeneration/remodeling during menstrual phases, likely reflecting the already observed FAP accumulation in various tissue remodeling processes. This hypothesis seems to be supported by other literature evidence: high endometrial ^68^Ga-FAPI uptake has been reported in the post-partum/lactation period and in younger women [47], with decreasing activity observed with increasing age, likely due to reduced endometrium FAP expression in the post-menopausal women [4]. In addition, regarding ^68^Ga-FAPI breast uptake, Backhaus et al. evaluated 19 women affected by BC, who performed a dedicated-breast PET/MR [39]. The authors reported a relationship between breast density (defined as the proportion of epithelial and/or fibroglandular tissue to fatty tissue) and tracer uptake [48]. In particular, a positive correlation between ^68^Ga-FAPI activity of breast background (i.e., activity of healthy breast tissue, measured in the contralateral breast or in a healthy part of the affected breast) and both higher background parenchymal enhancement and fibroglandular tissue density at MR was found. An inverse correlation was also found between healthy breast tissue activity and patients’ age, with decreasing uptake values with increasing age, likely reflecting the physiological breast density reduction with aging (in favor of fatty replacement). This finding is likely an expression of changes in breast density as an effect of physiological hormonal stimulation, with density reduction during aging due to the physiological reduction of reproductive hormones levels. Breast density can change in response to hormonal stimuli not only during normal aging, but also during pregnancy (reflecting a great amount of circulating estrogen and progesterone), during lactation (reflecting high prolactin production), and during exogenous hormone replacement [48]. This is in line with functional breast patterns observed in several case reports: Wang et al. reported intense and diffuse uptake of ^68^Ga-FAPI-04 in both breasts of a female patient, consistent with her menstrual history (in the ovulation period) [46]; Sonni et al. observed a symmetric, diffuse and bilateral breast ^68^Ga-FAPI-46 uptake in a young woman with cervical cancer who performed PET/CT during hormonal stimulation with gonadotropin injections for oocyte retrieval, hypothesizing hormonal stimulation as the cause of changes in cellular FAP expression or in the number of FAP-expressing fibroblasts in hormone-sensitive tissues [49]; a bilateral ^68^Ga-FAPI-46 breast uptake observed in the post-partum period in a breast-feeding woman was attributed to an increase in breast density and FAP expression as an effect of prolactin releasing (during lactation), that promotes secretory changes in the mammary glands [47].

#### 3.2.5. ^68^Ga-FAPI PET Diagnostic Performance: Whole-Body Disease Assessment and Comparison with ^18^F-FDG

The diagnostic performance of ^68^Ga-FAPI PET/CT was compared to the standard-of-care ^18^F-FDG PET/CT in several case reports and in four main research studies (Table 2), evaluating a cohort of BC patients ranging from 14 to 48 patients [6,25,29,32]. From a diagnostic point of view, even with some discordant findings, the prevalent evidence seems to state the superiority of ^68^Ga-FAPI in detecting nodal and distant metastatic localizations, with ^68^Ga-FAPI often identifying more lesions than ^18^F-FDG. This improved metastases’ detection lies in both a higher ^68^Ga-FAPI lesions’ uptake and in an overall lower background uptake in normal tissues than ^18^F-FDG (such as in brain, liver, bone and intestinal tract, with the exception of skeletal muscles). In particular, this favorable low ^68^Ga-FAPI background activity allows one to detect lesions otherwise masked by physiological high ^18^F-FDG uptake. In detail, regarding nodal metastatic involvement, an overall higher ^68^Ga-FAPI sensitivity was reported, due to higher nodal uptake and TBR over ^18^F-FDG [6], so supporting the possibility to identify a major number of metastatic nodes [32]. However, the higher FAPI radiotracer uptake may potentially lead to more false-positive nodal results, with a lower specificity of ^68^Ga-FAPI compared to ^18^F-FDG [21]. When considering distant metastases in liver and bone (representing the commonest target organs of BC metastatic involvement), ^68^Ga-FAPI showed lower background activity than ^18^F-FDG in both districts and higher bone lesions’ uptake intensity [6,29,32]. Conversely, ^68^Ga-FAPI uptake in liver metastases was not significantly higher in all the comparison studies [6,29,32]. However, the concordantly reported lower hepatic background activity with ^68^Ga-FAPI may allow the identification of more liver metastases than ^18^F-FDG due to a better detection and delineation of hepatic lesions [6,21,24,38], so overcoming the potential ^18^F-FDG limited sensitivity, especially in small-sized liver metastases. Similarly to liver, the high ^18^F-FDG brain uptake, which reflects its physiologically high glucose metabolism, negatively influences the detection of primary brain tumors and metastases because of masking the lesion signal. Regarding BC, some studies demonstrated that brain metastases showed lower ^68^Ga-FAPI uptake values than ^18^F-FDG, but a higher TBR due to a very low ^68^Ga-FAPI uptake in normal brain tissue, so resulting in an overall better image contrast of cerebral lesions [21,24,25,29]. Moreover, ^68^Ga-FAPI PET/CT allowed a clear visualization of leptomeningeal metastases, which cannot be properly studied with ^18^F-FDG PET/CT [24]. Diagnostic advantages of ^68^Ga-FAPI over ^18^F-FDG have also to be considered regarding the detection of visceral metastases, including peritoneal carcinomatosis. Indeed, the accuracy of ^18^F-FDG PET/CT for detection of peritoneal carcinomatosis is knowingly limited by the physiological tracer accumulation in the intestinal tract, a heterogeneous uptake in the intestinal wall due to peristaltic activity and a non-specific intestinal uptake in patients under oral anti-diabetic therapy [11,50]. Conversely, ^68^Ga-FAPI does not show non-specific intestinal uptake, resulting in very low rates of false-positive/false negative findings and in a better imaging contrast of the involved visceral peritoneum, mesentery and omenta [21]. With regard to the detection of metastatic lung involvement, discordant findings can be found. Indeed, if some authors reported that ^68^Ga-FAPI is comparable or even superior to ^18^F-FDG in lung evaluation [8,29,32], other authors demonstrated a higher ^18^F-FDG lesions’ uptake or no significant differences in normal lung parenchyma activity between the two radiotracers [6].

#### 3.2.6. ^68^Ga-FAPI PET Diagnostic Performance: False Positive and False Negative Findings

When interpreting ^68^Ga-FAPI PET images, especially considering the limited experience due to the very recent introduction of FAPI radiotracers, PET false positives and negatives have to be taken into account. Regarding false positives, increased ^68^Ga-FAPI uptake can be observed in inflammatory processes and post-surgical wound healing (in analogy to ^18^F-FDG). Indeed, in these benign conditions, ^68^Ga-FAPI is taken up by activated fibroblasts/myofibroblasts involved in inflammation-induced fibrosis/tissue remodeling [4,47]. Potential false positives have to be considered when observing foci of increased ^68^Ga-FAPI uptake in the breast: literature reports on foci of ^68^Ga-FAPI uptake appearing as suspicious for malignancy into breast parenchyma, then correctly diagnosed at histopathology as benign intra-mammary lymphoid tissue [27], fibrosis/scarring areas [2] or inflammatory changes at the biopsy site. In this regard, previous studies have reported on intense ^68^Ga-FAPI uptake in areas of unspecific breast fibrosis induced by inflammation after surgery or radiation therapy, so requiring careful PET images’ interpretation [24,29]. The occurrence of benign lesions presenting with high ^68^Ga-FAPI uptake must be carefully considered also on a whole-body evaluation. In this regard, the study by Zheng et al. specifically evaluated benign lesions showing increased ^68^Ga-FAPI-04 uptake in 182 patients with different tumor types (BC = 16) [31]. The authors reported increased ^68^Ga-FAPI uptake in various non-malignant processes, including acute or chronic inflammatory/infective diseases (such as osteoarthritis, enthesopathy, periodontitis, mastoiditis, chronic pancreatitis, esophagitis, appendicitis, prostatitis, lymphadenitis, hemorrhoids, pneumonia, tuberculosis), post-operative changes and sites of fractures (occurring 1 month to 4 years before PET). Moreover, although the SUVmax of benign lesions was generally lower than that of malignant tumors, a significant SUVmax overlap was observed between malignant and benign lesions. Other authors reported on incidental/occasional false-positive ^68^Ga-FAPI PET findings corresponding to additional benign conditions, such as elastofibroma dorsi [2], benign soft-tissue tumors, hepatic focal nodular hyperplasia [39] and bone cysts [40], or related to diseases such as myelofibrosis, liver cirrhosis and granulomatous disease that are pathologically characterized by a chronic activation of a fibrotic reaction, so implying an increased ^68^Ga-FAPI activity [21]. In order to summarize the commonest unspecific or non-malignant ^68^Ga-FAPI PET/CT uptake findings, Kessler et al. published a pictorial analysis derived from 91 patients: the most frequent pitfall findings were associated with degenerative processes at the joints and vertebral bones, followed by inflammatory processes or physiological/unspecific transient tracer retention at head–and-neck sites (teeth, salivary glands, oral or nasal mucosa); moreover, unspecific and stable increased ^68^Ga-FAPI uptake was also found in larger muscle groups with predilection for quadriceps femoris, latissimus dorsi and triceps muscle [4]. On the other hand, regarding the occurrence of false negatives findings, it is worth noting that ^68^Ga-FAPI PET/CT sensitivity can be suboptimal in some cases. For example, a low sensitivity for detecting bone metastasis was reported in one BC patient performing PET/CT for staging, probably due to low FAP expression in early osteogenic bone lesions [30]. Other possible false-negative findings can be related to factors knowingly affecting lesion detectability, such as a very small lesions’ size, as reported in millimetric pulmonary nodules or millimetric satellite BC lesions with a maximum diameter of 3 to 5 mm (due to the partial volume effect, which implies an underestimation of real tracer uptake in lesions with size below the PET system spatial resolution) [39], or a reduced TBR, as reported in cirrhotic patients with coexistent neoplastic liver lesions (due to the aforementioned diffuse increased background ^68^Ga-FAPI uptake caused by liver fibrosis) [31].

### 3.3. Theranostic Application

#### 3.3.1. Clinical and Methodological Studies’ Characteristics

Table 3 reports the main clinical and methodological characteristics of the six studies evaluating theranostic FAPI-radioligands application in overall 16 metastatic BC patients. Regarding study design, 4/6 were prospective and the others were case reports [25,44]. Among all studies, 3/6 regarded only BC patients and 3/6 a mixed population of various cancer patients (up to a maximum of 5 BC patients in each study). In 15/16 patients, the histotype was not reported; 1/16 had an intraductal carcinoma. The most frequent sites of distant metastases were lung, bone, liver and lymph nodes. All patients had a metastatic progressive disease: previous lines of treatment (radiotherapy, chemotherapy, hormonal therapy) were reported in 3/6 studies [41,43,44]; one patient received radioembolization on liver metastases and ^177^Lu-labeled HER2/diphosphonates [43]. Inclusion criteria for FAPI-RLT were reported in all studies, consisting in adequate FAPI expression in pre-treatment FAPI-based functional imaging (PET/CT or SPECT/CT); conversely, exclusion criteria were reported only in one paper [41].

#### 3.3.2. Pre- and Post-Treatment Imaging

Pre-treatment imaging was performed as follows: in 5/16 patients with planar whole-body and SPET/CT scintigraphic images 60 min after injection of a diagnostic dose (370 MBq) of ^177^Lu-FAPI-46 [41]; in others 5/16 patients with ^68^Ga-DOTA.SA.FAPI PET/CT [25,42]; in 6/16 patients with ^68^Ga-FAPI-04 or ^68^Ga-FAP-2286 PET/CT, respectively [9,43,44]. Diagnostic criteria for defining patients as “positive” at FAPI images were clearly defined in only one study [41]: the presence of increased tracer uptake findings at early or delay images or at both; a visual score was established with four degrees of lesion uptake (absent, mild, moderate and intense). In all studies, therapy with radiolabeled-FAPI was approved based on intense tracer uptake at pre-treatment imaging in at least one lesion [41], or in most lesions. Semiquantitative parameters were also used in 2/6 studies as a cut-off for treatment: SUVmax > 3 [42], or a SUVmax target/liver ratio > 3 [43]. Post-treatment images were obtained with planar whole-body and SPET/CT scintigraphic images from 3 h to 7 days after therapy.

#### 3.3.3. FAPI Radioligand Therapy

In the 6 studies, different radioligands were used for treatment: 5/16 patients were treated with ^177^Lu-FAPI-46; 4/16 with ^177^Lu-DOTA.SA-FAPi; 4/16 with ^177^Lu-FAP-2286; 1/16 with ^177^Lu-DOTA.GA.(SA.FAPI)2; 1/16 with ^90^Y-FAPI-46; and 1/16 with ^90^Y-FAPI-04, respectively. Each patient received from one to four treatments, with an interval of 4 to 8 weeks. Administered activities ranged from 2.9 to 9.9 GBq per cycle, decided on an empirical basis: injected activity was adapted according to the patient’s clinical condition, hematologic and renal function, but also the tumor distribution (i.e., in case of red marrow involvement, when a pre-existing grade 2 anemia needed a reduction of the administered activity). Injection modalities are reported in two studies [25,41]: A tracer was administered in 10 min under steroid cover. Dosimetry information about dose to target and normal tissues is achievable in only one study about three patients treated with ^177^Lu-DOTA.SA.FAPI [42]. Absorbed dose for whole-body was 1.10 × 10^−2^ +/− 1.72 × 10^−3^ Gy/GBq, 1.15 × 10^−1^ +/− 9.02 × 10^−3^ Gy/GBq for liver, 6.18 × 10^−1^ +/− 1.54 × 10^−2^ Gy/GBq for kidneys, 3.99 × 10^−3^ +/− 2.18 × 10^−4^ Gy/GBq for spleen, 9.84 × 10^−4^ +/− 2.58 × 10^−4^ Gy/GBq for bone marrow, and a median value of 6.03 × 10^−1^ Gy/GBq (IQR 2.30 × 10^−1^–1.81 × 10) for target lesions, respectively. Adverse events were evaluated in 14/16 patients: only five of them presented pain worsening and hematological complications level G3 [43]. Clinical response was reported in 11/16 patients: three of them obtained reduction of pain and therapy, six were stable and two had progression. Radiological response (RECIST 2.0) to treatment was achievable in 8/16 patients: four presented stable disease and the others, progression of disease. Overall survival was achievable in ten patients with a follow-up of 4.5 to 18 months: 5/5 were alive 4.5 months after treatment [41], 3/4 were alive 18 months after treatment [43], and one was still alive 11 months after treatment [44]. The different therapeutic efficacy of FAPI-radiopharmaceuticals could be related to tumor retention time and consequent absorbed dose. ^177^Lu-FAP-2286 has a median retention time of 44 h in bone metastases and 32 h in liver lesions (with means an absorbed dose of 3.0 and 0.4 Gy/GBq, respectively), which is eight/nine times higher than ^177^Lu-FAPI-46. Similarly, ^177^Lu-DOTAGA (SA.FAPI)2 has a median retention time of 86.6 h in malignant lesions, with a median absorbed dose of 6.70 Gy/GBq, much longer than 14 h and 0.603 Gy/GBq of ^177^Lu-DOTA-SA-FAPi.

## 4. Discussion

This narrative review is focused on the role of radiolabeled FAPI in patients with breast cancer, aiming to summarize the main evidence on this specific cancer. The first application of FAPI tracers for both diagnostic and therapeutic purpose in BC patients dates back to 2018, from the pioneering experience of a German research group [8,9,10]. Data from our review demonstrated that, from this starting point, literature evidence about the use of FAPI tracers in BC patients has rapidly grown, to a major extent in the diagnostic use of ^68^Ga-labeled FAPI PET/CT both at staging or restaging, but with increasing reports also in the theranostics perspective.

### 4.1. ^68^Ga-FAPI PET Diagnostic Studies: Clinical and Methodological Studies’ Characteristics

When considering all the selected diagnostic studies, a total of 175 patients with BC were evaluated, with a prevalent prospective enrollment. A small sample size was considered in each study (with only one study including more than 20 patients), so supporting the need of further FAPI studies in larger BC samples. However, it has to be considered that such still limited clinical evidence is likely depending on the very recent development of FAPI radiotracers. Among the included studies, heterogeneity in some clinical and methodological aspects was observed, such as the use of different FAPI ligands, also in the same study; nevertheless, since they share a common substrate, these FAP-targeting ligands seem to provide comparable diagnostic performance [6]. Moreover, intra- or inter-studies differences were observed such as a miscellaneous of early and advanced/metastatic stages or a mixed population of treatment-naive and previously treated patients (e.g., at restaging, with inoperable BC, or with metastatic or recurrent disease). Such heterogeneity may influence the tumor uptake of ^68^Ga-FAPI, reflecting changes of CAFs “burden” during the disease course or under the effect of previous therapies. In particular, it was suggested that BC cells can recruit normal stromal fibroblasts and, under conventional chemotherapy regimens, metabolically and phenotypically induce the transformation of these cells into CAFs (which support BC cells progression), acting as a treatment-resistance mechanism [51]. Based on this assumption, it is so expected that including both untreated and pre-treated BC patients within the same study population and/or across different studies can influence the measured ^68^Ga-FAPI uptake values in tumor lesions.

### 4.2. ^68^Ga-FAPI PET Diagnostic Performance: Primary BC Lesion

When considering the ^68^Ga-FAPI diagnostic performance in the primary BC lesion, tissue changes induced by previous treatments (systemic therapies or local radiation therapy) may partly explain the observed variations in tracer uptake among studies. Differences in histological features may account for an additional part of the reported variations in the primary BC lesion activity, with ductal and HER-2 positive tumors showing a trend for a greater uptake than lobular and the HER-2 negative counterpart. However, ^68^Ga-FAPI demonstrated an overall high tumor uptake in each tumor subtype, as well as in both high- and low-grading tumors, so resulting as a well-performing diagnostic tracer in any BC patient. This finding is supported by a previous study demonstrating, by means of immunohistochemistry, that FAP is well expressed in BC, regardless of intrinsic histological features [52]. The high ^68^Ga-FAPI avidity across all histological and molecular BC subtypes emerges as a main diagnostic advantage over ^18^F-FDG, especially for overcoming the limit of ^18^F-FDG sensitivity in tumors characterized by low glucose metabolism (such as lobular and Luminal A cancers). Indeed, it is well known that ^18^F-FDG uptake in BC depends on many factors such as tumor grading (low-grading lesions showing lower uptake), histologic types (lobular carcinomas showing lower uptake than invasive ductal carcinomas), hormonal receptor status (lower uptake in estrogen-positive and progesterone-positive well-differentiated tumors than hormonal receptor-negative tumors) and molecular subtypes (Luminal A showing lower uptake than others, due to a generally low-grading and low mitotic activity) [53,54]. From a practical point of view, these observations support the future potential use of ^68^Ga-FAPI PET/CT as complementary imaging, especially in BC with low ^18^F-FDG avidity. Interestingly, in the Luminal A subtype, the observed high ^68^Ga-FAPI uptake (mean SUVmax: 10; FDG-FAPI ratio: 0.3) [32] may find a biological substrate when considering that ^68^Ga-FAPI uptake seems to vary according to different expression of CAF-related proteins in different BC stroma types (fibrous or adipose). Indeed, FAP is mostly secreted by adipose stromal CAFs in tumor microenvironment, which are predominant in the Luminal A molecular subtype (while the fibrous stroma is prevalent in HER-2, Luminal B and triple-negative subtypes) [29,51]. In detail, among various hypotheses about the CAFs origin, it has been supposed that tumor-surrounding adipocytes are the main precursors of CAFs in BC, with BC cells affecting adjacent adipocytes, with a consequent down-regulation of their lipid content and up-regulation of fibroblast markers (including FAP) [51]. Finally, the high diagnostic performance of ^68^Ga-FAPI PET/CT for primary tumor detection lies both in a higher BC tracer uptake and in a favorable, physiological low TBR (due to a generally low activity in the surrounding normal breast parenchyma), so being able to guarantee an optimal tumor delineation, generally with better sensitivity than ^18^F-FDG. Nevertheless, when interpreting PET images in hormone-sensitive organs like the breast, the influence/interference of endogenous or exogenous hormonal stimulation on ^68^Ga-FAPI uptake has to be considered with caution as a potential diagnostic pitfall [6,46,47,49]. In practical management, it was stated that the hormonal-induced diffuse and increased tracer activity in normal breast parenchyma may reduce the detectability of BC lesion, and it has been suggested to preferably avoid performing a PET scan during the ovulation period in pre-menopausal patients.

### 4.3. ^68^Ga-FAPI PET Diagnostic Performance: Whole-Body Disease Assessment

When overall evaluating the diagnostic application of ^68^Ga-FAPI PET/CT, some clear advantages of ^68^Ga-FAPI over the standard-of-care ^18^F-FDG can be identified, mostly in methodological aspects: (1) ^68^Ga-FAPI has a fast clearance and shows lower off-target accumulation compared to ^18^F-FDG, so possibly reducing radiation doses [6]; (2) diagnostic ^68^Ga-FAPI images can be obtained just 10 to 30 min post-injection, due to its kinetics (versus 60 min p.i. when using ^18^F-FDG); (3) ^68^Ga-FAPI biodistribution is independent from the resting or fasting state and not influenced by muscles activity, since its accumulation is not influenced by blood glucose levels or the patient’s movement, in contrast to ^18^F-FDG [6]; (4) no ^68^Ga-FAPI uptake in case of adipose brown tissue activation is expected, so avoiding potential interference on images evaluation. From a practical point of view, these advantages may especially improve patients’ care and comfort: patient candidates to ^68^Ga-FAPI imaging do not need to fast or to check blood glucose levels before tracer injection (with a greater impact on management of diabetic ones); a shorter waiting time after injection turns into a shorter duration time of the overall PET/CT procedure. When “comparing” the diagnostic performance of ^68^Ga-FAPI and ^18^F-FDG, it has to be considered that the different radiotracer characteristics imply an intrinsic different ability for detecting a malignant lesion. Indeed: (1) the two tracers are markers of different targets in a tumor lesion: tumor stroma cells in one case, and properly tumor cells in the other; (2) lesions’ uptake reflects different biological mechanisms for each of them: stromal FAP expression and cancer glucose metabolism, respectively; (3) tumor cells generally account for less than 10% of the total cancer tissue, whereas the tumoral stroma accounts for all the rest. In particular, this latter observation implies that the ^18^F-FDG detection rate is more variable, depending not only on tumor histology and aggressiveness, but also on the number of malignant cells in the lesion. Given that, data from available comparative studies (although still derived from limited patients’ cohorts) seem to state the superiority of ^68^Ga-FAPI for detecting nodal and distant metastases, overcoming some well-known ^18^F-FDG limitations. In particular, ^68^Ga-FAPI was generally able to identify more lesions than ^18^F-FDG due to higher lesions’ uptake and/or a lower background tissue activity, with a major impact for detecting otherwise occult lesions in organs characterized by a high to moderate physiological or unspecific ^18^F-FDG uptake (such as liver, brain, bone, intestinal tract and peritoneal tissue). Given that lesions with a size around or below the spatial resolution of the PET system can be detected only if presenting a sufficiently intense tracer uptake and higher than surrounding tissue, the favorable ^68^Ga-FAPI target-to-background ratio (due to both high specific uptake and low background activity) explains its ability to disclose additional very small malignant lesions, i.e., with a smaller size than those detectable by ^18^F-FDG [21,24]. Apparently in contrast to the above reported comparative results, it is worth mentioning the study by Ballal et al., performing a head-to head comparison between ^68^Ga-DOTA.SA.FAPi and ^18^F-FDG PET/CT in patients with various cancers, including 20 BC patients [25]. On a patient-based analysis, this study is the first to show an overall similar diagnostic accuracy between the two tracers, with ^68^Ga-DOTA.SA.FAPi imaging closely matched to the standard-of-care ^18^F-FDG [25]. In detail, they reported a complete concordance between the two tracers for detecting liver and skeletal metastases and pleural thickening (with no significant differences in the measured uptake values), and only some discordant results with regard to nodal metastases (with both false-negative ^68^Ga-DOTA.SA.FAPi and false-positive ^18^F-FDG cases), lung lesions (with ^68^Ga-DOTA.SA.FAPi superiority in some patients) and brain localizations (with ^18^F-FDG PET/CT failing to identify cerebral metastases in two patients). Although this study does not show a great difference in the diagnostic performance between the two tracers, it seems not fully comparable with the other reported head-to-head comparative studies in BC patients that, conversely, found a superior detection rate of ^68^Ga-FAPI. Indeed, Ballal et al. compared the accuracy of the two tracers mostly considering all metastatic lesions as one group, regardless of the primary tumor of origin, so limiting the extraction of comparative data specifically derived from BC population; moreover, the observed equivalent ^18^F-FDG accuracy may be attributed to the enrollment of oncologic patients with a prevalence of ^18^F-FDG-avid histotypes, whereas previous authors enrolled mixed oncologic patients with a prevalence of low ^18^F-FDG-avid histotypes, so implying an easier chance of ^68^Ga-FAPI diagnostic superiority [21].

### 4.4. ^68^Ga-FAPI PET Diagnostic Performance: False Positive and False Negative Findings

Despite the overall diagnostic superiority of ^68^Ga-FAPI PET/CT demonstrated by most comparative studies, we have to mention the chance of some ^68^Ga-FAPI false negative lesions. On the other hand, it is also important to notice that ^68^Ga-FAPI should not be considered a fully tumor-specific PET tracer due to the occurrence of false positive findings, that are more frequently reported in literature than false negative ones. In particular, increased ^68^Ga-FAPI uptake at benign sites is mainly related to activated fibroblasts/myofibroblasts in inflammatory processes and post-surgical wound healing [4,47]: if FAP in normal stromal fibroblasts can be considered substantially absent or only expressed at low levels, up-regulation of FAP expression in activated normal fibroblasts seems to be modulated in response to non-tumoral stimuli, such as in inflammation-induced fibrosis/tissue remodeling [11,31]. In this regard, as happens with ^18^F-FDG PET imaging, ^68^Ga-FAPI PET images’ interpretation may be challenging not only in whole-body assessment, but also at the breast site: the differential diagnosis between residual/recurrent disease and post-operative/post-radiation inflammatory reaction can be problematic [24], also considering that tissue remodeling may last for a long time after tissue damage [31]. Therefore, in all doubtful cases, accurate knowledge of clinical data and integrated evaluation of morphological patterns at co-registered CT images may help readers to differentiate active benign lesions from true malignancy.

### 4.5. FAPI Radioligand Therapy

Radiolabeled-FAPI is both a diagnostic and a possible future therapeutic agent in the oncological setting, so conferring a theranostic role [6]. The development of FAPI radiotracers has introduced the theranostic concept also into the BC field, increasing the possibility of more personalized treatments based on individual and tumor characteristics. However, the studies nowadays published about FAPI therapy in BC are only six, two of them representing case reports. Moreover, these papers concern a very heterogeneous population of cancer patients. All the studies mainly emphasize the feasibility and the safety of FAPI radioligand therapy, with only five severe adverse events, but on a very small number of cases. All the patients were in an advanced stage of disease, with multiple metastases and many previous treatments carried out. The indication for treatment has been established on the basis of visual evaluation of diagnostic images: in only two studies, semiquantitative parameters have been assessed. Moreover, the authors have used many types of FAPI pharmaceuticals, labelled with ^177^Lu or ^90^Y, without any standardization or dosimetry approach. Activities to be administered, number of cycles and interval between them were empirically decided. Finally, PFS was not calculated, and the OS was established after a very short follow-up time.

## 5. Conclusions

From our review, despite heterogeneity in some clinical and methodological aspects among the evaluated studies and an overall small sample size, ^68^Ga-FAPI PET/CT emerges as a valuable diagnostic tool in BC patients both at staging and restaging, also demonstrating several technical advantages over the standard-of-care ^18^F-FDG. Beside a favorable lesion-to-background ratio, ^68^Ga-FAPI demonstrates an overall high tumor uptake across all histological and molecular BC subtypes and an overall higher detection rate for metastatic involvement than ^18^F-FDG. These results support, in the next future, the application of radiolabeled FAPI PET/CT as a complementary diagnostic tool to ^18^F-FDG, especially in patients with: (1) BC histotypes characterized by well-known low ^18^F-FDG-avidity (such as lobular and Luminal A cancers); (2) inconclusive ^18^F-FDG PET/CT findings at staging or restaging (e.g., lesions with absent or minimal tracer uptake or metabolically not distinguishable from adjacent background activity); (3) negative ^18^F-FDG imaging, but highly suspected disease recurrence (e.g., based on elevated tumor markers values or clinical symptoms); (4) loco-regional or oligo-metastatic disease at ^18^F-FDG PET/CT. ^68^Ga-FAPI seems to play a major clinical impact in this latter scenario due to its potential to identify unexpected or additional metastatic lesions, so impacting on both staging and treatment management. Indeed, in those patients with apparently confined BC (and so, candidates for loco-regional therapy), additional ^68^Ga-FAPI PET/CT could identify unexpected metastases, so upstaging patients from M0 to M+ and changing the treatment planning. In ^18^F-FDG oligo-metastatic patients, too, additional ^68^Ga-FAPI PET/CT could point out a more extensive disease spread by detecting additional metastases, so leading to changes in the planned treatment strategy.

## 6. Future Directions

From the analyzed data, the need for conducting larger comparative prospective studies to further define the diagnostic accuracy of radiolabeled FAPI PET/CT, especially in more homogeneous BC populations (such as with the same clinical disease stage, only untreated patients, all examined for suspected recurrence), emerges as one of the main future perspectives. Moreover, given the promising but still clinically limited results of FAPI radioligand therapy in BC patients, future prospective studies with a large cohort of patients and with standardized procedures are needed. Finally, there is a general agreement on the negative prognostic role of CAFs in various solid tumors (including BC), with high FAP expression associated with a more aggressive tumor behavior, disease progression and treatment resistance [2,11,51,55,56]. Therefore, ^68^Ga-FAPI studies aiming to improve the understanding of the role of FAPI as a prognostic indicator in BC patients represent an interesting future direction of clinical research.

## Figures and Tables

**Table 1:** Clinical and methodological characteristics of FAPI PET/CT diagnostic studies (*n* = 27).

First Author	Year of Publication	Journal	Country	Study Design	Study Population	Indication for FAPI Imaging	Patients with BC (*n*)	PET Modality	FAPI Tracer	Activity	Scan Delay (p.i.)	FOV
Loktev A [10]	2018	JNM	Germany	n.s.	Mixed tumors	Proof of concept	1	PET/CT	^68^Ga-FAPI-02	222–312 MBq	10 min, 1 h and 3 h	Vertex–upper thigh
Giesel FL [8]	2019	JNM	Germany	R	Mixed tumors	Unmet diagnostic challenge or FAP-radioligand therapy suitability	2	PET/CT	^68^Ga-FAPI-02, ^68^Ga-FAPI-04	122–336 MBq	1 h and 3 h	Vertex–upper thigh
Loktev A [12]	2019	JNM	Germany	R	Mixed tumors	Proof of concept	2	PET/CT	^68^Ga-FAPI-04, ^68^Ga-FAPI-21, ^68^Ga-FAPI-46	210–267 MBq	10 min, 1 h and 3 h	Vertex–upper thigh
Kratochwil C [11]	2019	JNM	Germany	R	Mixed tumors	Unmet diagnostic challenge	12	PET/CT	^68^Ga-FAPI-04	122–312 MBq	1 h	Vertex– midthigh
Meyer C [20]	2020	JNM	USA, Germany	R	Mixed tumors	Biodistribution and dosimetry	1	PET/CT	^68^Ga-FAPI-46	214–246 MBq	10 min, 1 and 3 h	Vertex– upper thigh
Chen H [21]	2020	EJNMMI	China	P	Mixed tumors	Staging or restaging	1	PET/CT	^68^Ga-FAPI-04	1.8–2.2 MBq/kg	1 h	Head–upper thigh
Pang Y [22]	2020	Clin Nucl Med	China	P	BC	Restaging	1	PET/CT	^68^Ga-FAPI (n.o.s.)	n.s.	n.s.	Vertex–midthigh
Shi X [23]	2021	EJNMMI	China	P	Mixed tumors	Hepatic nodules characterization	1	PET/CT	^68^Ga-FAPI-04	96–260 MBq	1 h	Vertex–upper thigh
Chen H [24]	2021	EJNMMI	China	P	Mixed tumors	Inconclusive 18F-FDG findings	4	PET/CT	^68^Ga-FAPI-04	1.8–2.2 MBq/kg	1 h	Vertex–upper thigh
Ballal S [25]	2021	EJNMMI	India, Germany, Chile	P	Mixed tumors	Biodistribution, pharmacokinetics and dosimetry	20	PET/CT	^68^Ga-DOTA.SA.FAPi	59.2–296 MBq	1 h	Vertex–midthigh
Zhao L [26]	2021	EJNMMI	China	R	Mixed tumors	Suspected peritoneal carcinomatosis	1	PET/CT	^68^Ga-FAPI-04	1.8–2.2 MBq/kg	1 h	Vertex–upper thigh
Gündoğan C [27]	2021	Clin Nucl Med	Turkey	P	BC	Staging	1	PET/CT	^68^Ga-FAPI-04	n.s.	n.s.	Vertex–midthigh
Can C [28]	2021	Clin Nucl Med	Turkey	P	BC	Staging	1	PET/CT	^68^Ga-FAPI-04	n.s.	n.s.	Vertex–upper thigh
Kömek H [29]	2021	Ann Nucl Med	Turkey	P	BC	Staging or restaging	20	PET/CT	^68^Ga-FAPI-04	2 MBq/kg	1 h	Vertex–midthigh
Dendl K [6]	2021	EJNMMI	Germany, South Africa	R	Mixed tumors	Unmet diagnostic challenge or FAP-radioligand therapy suitability	14	PET/CT	^68^Ga-FAPI-02, ^68^Ga-FAPI-04, ^68^Ga-FAPI-46, ^68^Ga-FAPI-74	52–325 MBq	1 h	Vertex–midthigh
Zheng S [30]	2021	EJNMMI	China	P	BC	Staging	1	PET/CT	^68^Ga-FAPI (n.o.s.)	n.s.	n.s.	Head–upper thigh
Zheng S [31]	2021	Ann Nucl Med	China	R	Mixed tumors	Staging or restaging	16	PET/CT	^68^Ga-FAPI-04	3.7 MBq/kg	30–60 min	Head–upper thigh
Elboga U [32]	2021	Ann Nucl Med	Turkey	R	BC	Staging or restaging	48	PET/CT	^68^Ga-FAPI-04	2 MBq/kg	1 h	Vertex–midthigh
Wu J [33]	2021	Front Oncol	China	R	Mixed tumors	Bone metastases detection	1	PET/CT	^68^Ga-FAPI-04	1.85–2.59 MBq/kg	65 ± 5 min	Skull base–midthigh
Kömek H [34]	2021	Mol Imaging Radionucl Ther	Turkey	n.s.	BC	Staging	1	PET/CT	^68^Ga-FAPI-04	n.s.	n.s.	Vertex–midthigh
Çermik TF [35]	2022	Clin Nucl Med	Turkey	P	Mixed tumors	Staging, restaging, treatment response evaluation	1	PET/CT	^68^Ga-FAPI-04	1.85 MBq/kg	1 h	Vertex–upper thigh
Xu T [36]	2022	Clin Nucl Med	China	P	BC	Staging	1	PET/CT	^68^Ga-FAPI-04	n.s.	n.s.	Vertex–midthigh
Shang Q [37]	2022	EJNMMI	China	P	BC	Staging	1	PET/CT	^68^Ga-FAPI-04	n.s.	n.s.	Head–upper thigh
Mona CE [2]	2022	JNM	USA	P	Mixed tumors	Biodistribution, immuno-histochemistry correlation	2	PET/CT	^68^Ga-FAPI-46	184 ± 3 MBq	63 ± 10 min	Vertex–midthigh
Wang Q [38]	2022	Clin Nucl Med	China	P	BC	Staging	1	PET/CT	^68^Ga-FAPI-04	n.s.	n.s.	Vertex–midthigh
Backhaus P [39]	2022	Radiology	Germany	R	BC	Staging, restaging after treatment	19	PET/CT, PET/MR	^68^Ga-FAPI-46	149 ± 48 MBq	79 min	n.s.
Gungor S [40]	2022	Clin Nucl Med	Turkey	P	BC	Restaging	1	PET/CT	^68^Ga-FAPI (n.o.s.)	n.s	n.s.	Vertex–midthigh

FAPI: fibroblast activation protein inhibitor; PET/CT: positron emission tomography/computed tomography; BC: breast cancer; p.i.: post-injection; FOV: field of view; n.s.: not specified; P: prospective; R: retrospective; n.o.s.: not otherwise specified; 18F-FDG: 18F-fluorodeoxyglucose; FAP: fibroblast activation protein; PET/MR: positron emission tomography/magnetic resonance.

**Table 2:** Comparison studies on the diagnostic performance of ^68^Ga-FAPI PET/CT and ^18^F-FDG PET/CT (*n* = 4).

First Author	Patients (*n*)	Age (Years)	^68^Ga-FAPI Activity	^18^F-FDG Activity	Time between ^68^Ga-FAPI and ^18^F-FDG Scans	PET Image Analysis	^68^Ga-FAPI >^18^F-FDG	^68^Ga-FAPI ≤ ^18^F-FDG
Kömek H [29]	20	32–65	2 MBq/kg	3.5–5.5 MBq/kg	1 week	Semiquantitative (^68^Ga-FAPI and ^18^F-FDG)	- Higher sensitivity in detecting primary BC (100% vs. 78.2% ^18^F-FDG)	- Similar specificity in detecting primary BC (100% ^18^F-FDG vs. 95.6% ^68^Ga-FAPI) - Not a statistically significant difference between the two tracers in SUVmax values of hepatic metastases (*p* > 0.05)
							- Significantly higher SUVmax values in primary BC, lymph nodes, lung and bone metastases (*p* < 0.05)	
							- Significantly higher TBR values in BC, hepatic, bone, brain and lung metastases (*p* < 0.05).	
							- Lower background activity and higher uptake in subcentimetric lesions	
							- Lower physiological uptake in liver, bone and brain	
Elboga U [32]	48	53.3 ± 11.7	2 MBq/kg	3.5–5.5 MBq/kg	Max 1 week	Semiquantitative (^68^Ga-FAPI and ^18^F-FDG)	- More lesions detected in breast, lymph nodes and bone	-
							- Higher SUVmax values in primary BC, lymph nodes, lung, liver and bone metastases	
							- Higher SUVmax values for ILC, but not statistically significant (due to the low number of ILC patients)	
							- Change in therapeutic approach of 12 pts defined as PD by ^68^Ga-FAPI imaging (reported as PMR, CMR or SD by ^18^F-FDG)	
							- Better assessment of lesions in the first month of post-chemotherapy period	
Ballal S [25]	20	30–66	59.2–296 MBq	185–370 MBq	1 week	Qualitative and semiquantitative (^68^Ga-FAPI and ^18^F-FDG)	- A remarkably higher SULpeak and SULavg brain metastases-to-normal brain ratio (due to lower brain parenchyma physiological uptake) - Outstandingly higher uptake values for Krukenberg metastases (*p* < 0.0001)	- Comparable results for detection of primary BC, with comparable SUL values between the two tracers - Similar diagnostic accuracy for both tracers
Dendl K [6]	14	59.5 (median)	52–325 MBq	251–300 MBq	12.5 days (median)	Semiquantitative (^68^Ga-FAPI and ^18^F-FDG)	- High uptake, resulting in sharp contrasts in primary and metastatic lesions	- Higher ^18^F-FDG uptake in lung metastases (13.7 vs. 6.6; *p* = 0.18)
							- Slight advantages of mean SUVmax in all metastatic lesions (8.2 vs. 7.8; *p* = 0.131)	
							- Favorable mean SUVmax in lymph nodes (7.1 vs. 6.3; *p* = 0.753), bone (10.1 vs. 7.4; *p* = 0.138) and liver metastases (5.9 vs. 5.1; *p* = 0.593)	
							- TBRs slightly advantageous in regional lymph nodes (31.9 vs. 27.4; *p* = 0.6) and, significantly, in distant metastases (13.0 vs. 5.7; *p* = 0.047)	

FAPI: fibroblast activation protein inhibitor; 18F-FDG: 18F-fluorodeoxyglucose; PET/CT: positron emission tomography/computed tomography; BC: breast cancer; SUV: standardized uptake value; TBR: tumor-to-background ratio; ILC: invasive lobular carcinoma; PD: progression disease; PMR: partial metabolic response; CMR: complete metabolic response; SD: stable disease; SUL: standardized uptake value corrected for lean body mass.

**Table 3:** Clinical and methodological characteristics of FAPI theranostic studies (*n* = 6).

First Author	Year of Publication	Journal	Country	Study Design	Patients with BC (*n*)	BC Clinical Setting	Previous Treatments	Therapeutic FAPI Tracer	Overall Activity (GBq)	Treatment Response	Adverse Events
Assadi M [41]	2021	CNM	Iran	P	5	Metastatic progressive disease	SUR + CHT + EBRT	^177^Lu-FAPI-46	1.85–12.95 (range)	3/5 SD 2/5 PD 3/5 ECOGPS stable 2/5 ECOGPS worsening PFS not evaluated OS >2.0–5.0 months	Worsening of pain (1/5 pts)
Ballal S [25]	2021	EJNMMI	India	R	1	Metastatic progressive disease	n.s.	^177^Lu-DOTA.SA.FAPi	3.2	Reduction of pain PFS and OS not evaluated	No AEs
Ballal S [42]	2021	Pharmaceuticals	India	P	4	Metastatic progressive disease	n.s.	^177^Lu-DOTA.SA.FAPI (3 pts) ^177^Lu-DOTAGA.(SA.FAPI) (1 pt)	2.96 n.s.	Clinical response in all pts (symptoms) PFS and OS not evaluated	No AEs
Baum RP [43]	2022	JNM	Germany, USA, Singapore	R	4	Metastatic progressive disease	EBRT, chemo-embolization, ^177^Lu-HER2-ligand,^177^Lu-diphosphonate, CHT, hormonal therapy	^177^Lu-FAP-2286	8.3–14-4 (range)	PD after 2 cycles PFS not evaluated 3 pts alive after 18 months from 1 FAPI-RLT cycle	Pain, anemia, leukocytopenia (G1, G2, G3)
Lindner T [9]	2018	JNM	Germany	P	1	Metastatic progressive disease	n.s.	^90^Y-FAPI-04	2.9	Reduction of pain PFS and OS not evaluated	n.s.
Ratke H [44]	2021	CNM	Germany	P	1	Metastatic progressive disease	Hormonal therapy, CHT, diphosphonates	^90^Y-FAPI-46	28.1	SD PFS not evaluated OS 11 months	n.s.

FAPI: fibroblast activation protein inhibitor; BC: breast cancer; P: prospective; R: retrospective; SUR: surgery; CHT: chemotherapy; EBRT: external beam radiotherapy; RLT: radioligand therapy; SD: stable disease; PD: progression disease; ECOGPS: Eastern Cooperative Oncology Group Performance Status; PFS: progression free survival; OS: overall survival; n.s.: not specified; AEs: adverse events; HER2: human epidermal growth factor receptor 2.

## Data Availability

No new data were created or analyzed in this study.
